# Particle Swarm Optimization Based Feature Enhancement and Feature Selection for Improved Emotion Recognition in Speech and Glottal Signals

**DOI:** 10.1371/journal.pone.0120344

**Published:** 2015-03-23

**Authors:** Hariharan Muthusamy, Kemal Polat, Sazali Yaacob

**Affiliations:** 1 School of Mechatronic Engineering, Universiti Malaysia Perlis (UniMAP), Campus Pauh Putra, 02600 Arau, Perlis, Malaysia; 2 Department of Electrical and Electronics Engineering, Faculty of Engineering and Architecture, Abant Izzet Baysal University, 14280 Bolu, Turkey; 3 Universiti Kuala Lumpur Malaysian Spanish Institute, Kulim Hi-TechPark, 09000 Kulim, Kedah, Malaysia; Beijing University, CHINA

## Abstract

In the recent years, many research works have been published using speech related features for speech emotion recognition, however, recent studies show that there is a strong correlation between emotional states and glottal features. In this work, Mel-frequency cepstralcoefficients (MFCCs), linear predictive cepstral coefficients (LPCCs), perceptual linear predictive (PLP) features, gammatone filter outputs, timbral texture features, stationary wavelet transform based timbral texture features and relative wavelet packet energy and entropy features were extracted from the emotional speech (ES) signals and its glottal waveforms(GW). Particle swarm optimization based clustering (PSOC) and wrapper based particle swarm optimization (WPSO) were proposed to enhance the discerning ability of the features and to select the discriminating features respectively. Three different emotional speech databases were utilized to gauge the proposed method. Extreme learning machine (ELM) was employed to classify the different types of emotions. Different experiments were conducted and the results show that the proposed method significantly improves the speech emotion recognition performance compared to previous works published in the literature.

## Introduction

Speech utterances of an individual can provide information about his/her health state, emotion, language employed and gender. Speech is the one of the most natural form of communication between the individuals. Understanding of an individual’s emotion can be useful for applications like web movies, electronic tutoring applications, in-car board system, diagnostic tool for therapists and call-center applications. Most of the existing emotional speech database contains three types of emotional speech recordings such as simulated, elicited and natural. Simulated emotions tend to be more expressive than real ones and most commonly used. For the elicited category, emotions are nearer to the natural database but if the speakers know that they are being recorded, then the quality will be artificial. Next, in natural category, all emotions may not be available and difficult to model because these are completely naturally expressed [1,2,3,4]. Most of the researchers have analyzed four primary emotions such as anger, joy, fear and sadness either in simulated domain or in natural domain. High emotion recognition accuracies were obtained for two-class emotion recognition (High arousal Vs Low arousal), but multi-class emotion recognition is still disputing. This is due to the following reasons: (a) which speech features are information-rich and parsimonious, (b) different sentences, speakers, speaking styles and rates, (c) more than one perceived emotion in the same utterance, (d)long-term/short-term emotional state.Several speech features have been successfully applied for speech emotion recognition and can be mainly classified into four groups such as continuous features, qualitative features, spectral features and non-linear Teager energy operator based features [1,2,3,4]. Various types of classifiers have been proposed for speech emotion recognition such as hidden Markov model (HMM), Gaussian mixture model (GMM), support vector machine (SVM), artificial neural networks (ANN) and k-nearest neighbor (kNN) classifier [1,2,3,4].

Although several research works have beenconducted in the field of speech emotion recognition, it is difficult to compare them directly due tothe inconsistency in the division of dataset, number of emotions used, number of emotional speech databases used, simulated/elicited/natural speech emotional speech databases used and the lack of uniformity in the presentation and computation of the results. Most of the researchers have often used 10-fold cross validation, conventional validation (one training set + one testing set and speaker-dependent emotion recognition and achieved excellent performance. Speaker-independent multi-class emotion recognition is still a challenging taskdue to the higher degree of overlap among the speech features, irrelevant, redundant and noisy speech features. To improve the discrimination ability of the speech features and to select an optimal set of features with a modicum or no loss of emotion recognition accuracy, new methods was proposed in this work. Various well-known speech features were extracted from the emotional speech and glottal signals. PSO is a population—based stochastic optimization method and several PSO variants have been proposed in the literaturefor function optimization, clustering and feature selection [5,6,7,8,9,10,11]. PSO based algorithms were proposed in this work, as it is very popular among researchers due to its simple mathematical operations, a small number of control parameters, quick convergence and ease of implementation [5,6,7,8,9,10,11]. PSO based clustering and wrapper based PSO were proposed to improve the discrimination ability of the extracted speech features and toenhance the accuracy of speaker-independent multi-class emotion recognitionby selecting only discriminative features respectively. First, PSO based clustering was applied on the extracted feature set to find the centriod or cluster centers. From the centers and means of the each feature, weights were calculated and multiplied with the original features to enhance their discrimination ability. From the weighted features, optimal feature set was found using the proposed wrapper based PSO in which three modifications were suggested. The proposed method has the following salient features: (1). Enhancement of discrimination ability of the extracted features using PSO based clustering; (2). Selection of optimal feature set thereby the performance of multi-class speech emotion recognition system has been improved.

## Related Works

In [12], authors have proposed non-linear dynamic based features, prosodic and spectral features and used SVM classifier to classify seven emotions using the speech samples of Berlin emotional speech database (BES). They have achieved an emotion recognition accuracy of 82.72% for female speakers and 85.90% for male speakers using 10-fold cross validation. Non-linear dynamic features and neural network classifier were used to classify three emotions (Neutral, fear and anger) and obtained a maximum emotion recognition accuracy of 93.78% for speaker dependent case [13]. Modulation based spectral features and multi-class SVM were used by the researchers in [14] to classify the seven classes of emotions and obtained a maximum emotion recognition accuracy of 85.60%. In [15], authors have used a combination of spectral excitation source features and auto-associative neural network and their emotion recognition accuracy was 82.16%. K. S. Rao et.al., have employed a combination of utterance-wise global and local prosodic features with SVM classifier and they obtained an emotion recognition accuracy of 62.43% [16]. In [17], authors have used LPCCs, formants and GMM classifier for the classification of seven emotions and the emotion recognition accuracy was 68%. Discriminative wavelet packet band power coefficients with Daubechies filter order of 40 and GMM classifier were used by Y. Li et. al., in [18] and obtained a maximum emotion recognition accuracy of 75.64%.Kotti M and Paternò Fhave proposed several low level audio descriptors and high level perceptual descriptorsand achieved a maximum emotion recognition accuracy of 87.7% under speaker independent case with Linear SVM [19]. MPEG-7 low level audio descriptors and SVM with radial basis function (RBF) kernel were used for the recognition of seven emotions and the emotion recognition accuracy was 77.88% [20]. In [21], Mel-frequency cepstral coefficients (MFCCs) and signal energy were computed as features. Correlation based feature selection with SVM-RBF kernel were used and this method was tested on the speech samples of Surry audio-visual emotional speech database (SAVEE). The emotion recognition accuracy was 79%. Intensity of energy, pitch, standard deviation, jitter and shimmer were extracted as features to classify the seven emotions using the audio samples of SAVEE database. They used *k*NN classifier and obtained a maximum emotion recognition accuracy of 74.39% [22]. Several speech features, linear discriminant analysis (LDA) based feature reduction and single component Gaussian classifier were employed to classify the seven emotions and achieved a maximum emotion recognition accuracy of 63% [23]. In [24], pitch, energy, duration and spectral based features were extracted and Gaussian classifier was used to classify seven emotions using the audio samples of SAVEE database. They achieved a maximum emotion recognition accuracy of 59.20%.

Though speech related features are widely used for speech emotion recognition, there is a strong correlation between the emotional states and features derived from glottal waveforms. Glottal waveform is significantly affected by the emotional state and speaking style of an individual [25,26,27,28,29,30,31,32]. Alexander I and Michael Shave investigated the effectiveness of glottal features derived from the glottal airflow signal in recognizing emotions. The average emotion recognition rate of 66.5% for all six emotions (Happy, Angry, Sad, Fear, Surprise and Neutral) and 99% for four emotions (Happy, Neutral, Angry and Sad) were achieved [25]. In [26,27,28], researchers have investigated the relationship between the emotional stages and the speech produced under stress, where glottal waveform was affected due to the excessive tension or lack of coordination in the laryngeal musculature. The effectiveness of the glottal features was analyzed in the classification of clinical depression by Moore et.al., [30,31]. In [32], authors have proposed the glottal flow spectrum as a possible cues for depression and near-term suicide risk and obtained 85% of the correct emotion recognition rate.Ling He et.al., have proposed wavelet packet energy entropy features for emotion recognition from speech and glottal signals with GMM classifier [33]. They achieved the average emotion recognition rates for BES database between 51% and 54%. In [34], prosodic, spectral, glottal flow, AM-FM features were utilized and a two-stage feature reduction was proposed for speech emotion recognition. The overall emotion recognition rates of 85.18% for gender dependent and 80.09% for gender independent were achieved using SVM classifier.

## Materials and Methods

**Fig 1:**
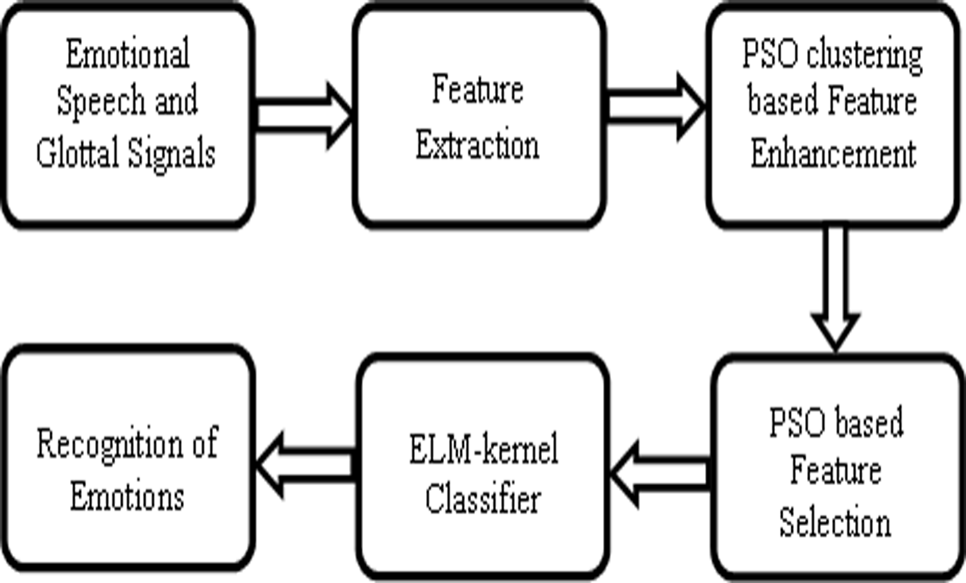
Proposed improved emotion recognition from emotional speech signals and its glottal waveforms.

This section describes the materials and methods used in this work. We have derived MFCCs, LPCCs, PLPs,gammatone filterbank outputs, timbral texture features, SWT based timbral texture features and relative wavelet packet based energy and entropy based features from emotional speech signals and its glottal waveforms. To extract the glottal and vocal tract characteristics from the speech waveform, inverse filtering and linear predictive analysis were used [41,42,43,44,45]. Feature selection and enhancement are the inevitable tasks in any pattern recognition problem. Higher degree of overlap among the features of different classes may degrade the performance of speech emotion recognition system. To decrease the intra-class variance and to increase the inter-class variance among the features, PSO based clustering was suggested. Raw featureswere called as weighted features after applying feature enhancement algorithm using PSO based clustering. Curse of dimensionality is a challenging issue in any pattern recognition problem. In the field of speech emotion recognition research, several filter, wrapper and embedded based feature selection methods are available in the literature to solve the issue of curse of dimensionality [35,36,37,38,39,40]. In this work, PSO based feature selection to select the discriminativeweighted features. Both raw and weighted features were subjected to different experiments to validate their effectiveness in speech emotion recognition. Extreme learning machine with RBF kernel was used as classifier to recognize different emotions. Fig. 1 shows the block diagram of the proposed improved emotion recognition system using PSO based feature enhancement and feature selection from emotional speech signals and its glottal waveforms.

### Emotional Speech Databases

In this work, three different emotional speech databases were used for emotion recognition to test the robustness of the proposed method.Berlin emotional speech database(BES) whic consists of speech utterances in German language. 10 professional actor/actresses were used to simulate 7 emotions (Anger—Ang, Boredom- Bor, Disgust-Dis, Fear- Fea, Happiness- Hap, Sadness—Sad, Neutral—Neu) [46]. Surrey audio-visual expressed emotion (SAVEE) database [24] is an audio-visual emotional database which includes seven emotion categories of speech and video signals (Anger—Ang, Disgust—Dis, Fear—Fea, Neutral—Neu, Happiness—Hap, Sadness—Sad and Surprise—Sur) from four native English male speakers aged from 27 to 31 years. 3 common, 2 emotion-specific and 10 generic sentences from 15 TIMIT sentences per emotion were recorded. In this work, only audio samples were utilized. Sahand Emotional Speech database (SES) was recorded at Artificial Intelligence and Information Analysis Lab, Department of Electrical Engineering, Sahand University of Technology, Iran [47]. This database contains speech utterances of five basic emotions (Neutral—Neu, Surprise—Sur, Happiness—Hap, Sadness—Sad and Anger—Ang) from 10 speakers (5 male and 5 female). 10 single words, 12 sentences and 2 passages in Farsi language were recorded which results in a total of 120 utterances per emotions. Table 1 gives the details of number of speech samples per emotion.

**Table 1:** Details of number of speech samples per emotion.

Databases	Emotions							
	Anger	Disgust	Fear	Neutral	Happiness	Sadness	Boredom	Surprise
BES	127	45	70	70	71	62	81	NA
SAVEE	60	60	60	120	60	60	NA	60
SES	240	NA	NA	240	240	240	NA	240

NA-Not Applicable

### Feature Extraction for Speech Emotion Recognition

In the design of a speech emotion recognition system, extraction of most informative features for efficiently characterizing different emotions is still an open issue. Researchers have commonly used short-term features, called frame-by-frame analysis. As the emotional speech signals were recorded at different sampling frequency, all the emotional speech samples were down-sampled to 8 kHz for convenience. From the recorded the emotional speech signals, the unvoiced portions between words were removed by segmenting the down-sampled emotional speech signals into non-overlapping frames with a length of 32 ms (256 samples) based on the energy of the frames. Frames with low energy were discarded and the rest of the frames (voiced portions) were concatenated and used for feature extraction [33]. Then the emotional speech signals (only voiced portions) are passed through a first order low pass filter to spectrally flatten the signal and to make it less susceptible to finite precision effects later in the signal processing [48]. The first order pre-emphasis filter is defined as

$$H(z)\;=\;1-\;a\;*\;z^{-1}0.9\leq a\leq 1.0$$ (1)

The commonly used *a* value is 15/16 = 0.9375 or 0.95 [48]. In this work, the value of *a* was set equal to 0.9375.Extraction of glottal flow signal from speech signal is a challenging task. In this work, glottal waveforms were estimated based on the inverse filtering and linear predictive analysis from the pre-emphasized speech waveforms.

#### Mel-frequency cepstral coefficients (MFCCs)

After pre-emphasis, the emotional speech signals/glottal signalsweresegmented into frames and windowed by Hamming window to minimize the signal discontinuities and spectral distortion. The fast Fourier transform (FFT)wasapplied to calculate the spectrum of the each frame, followed by Mel-scaled mapping to get the spectrum in Mel domain.The Mel-frequency scale is linear frequency spacing below 1 kHz and a logarithmic spacing above 1 kHz. Logarithmic Mel spectrum was obtained by taking the logarithm value of the signal after the Mel filters. Finally, MFCCs were generated by using discrete cosine transform (DCT) for a frame [49]. After obtaining the MFCCs for each frame, they were averaged over all frames.Totally, 48 MFCCs features which include 24 MFCCs from emotional speech signals and 24 MFCCs from emotional glottal signals were extracted.

#### Linear predictive cepstral coefficients (LPCCs)

36 LPCCs (18 LPCCs from emotional speech signals + 18 LPCCs from emotional glottal signals) were derived from LPC coefficients which are the coefficients of the Fourier transform representation of the log magnitude spectrum. The steps involved in the extraction of LPC coefficients are as follows: pre-emphasis, frame-blocking, windowing, autocorrelation analysis and conversion of autocorrelation coefficients to an LPC parameter set using Durbin’s method [48].The suitable value of LPC order from 8 to 16 was found and fixed as 12.After obtaining the LPCCs for each frame, they were averaged over all frames.

#### Gammatone filterbank outputs (GTFBOs)

Roy Patterson and his colleagues in 1992 originally proposed the Gammatone filterbank to provide a good approximation of human auditory filter and to visualize sound as a time-varying distribution of energy [50,51]. The pre-emphasised speech and glottal waveforms were fed into Gammatone filterbank. Twenty four Gammatone filterbank outputs were used in this work. A total of 48Gammatone filterbank outputs (24 for each emotional speech signals + 24 for each glottal waveforms) were derived for each emotional speech signals and its glottal waveforms.

#### Perceptual linear predictive (PLP) analysis

Itis a combination of short-term spectral analysis and LP analysis. It uses three basic concepts from the psychophysics of hearing concepts such as the critical-band spectral resolution, the equal-loudness curve and the intensity-loudness power law to derive an estimate of auditory spectrum. Finally, this auditory spectrum was approximated by using the auto-correlation method of all-pole modeling and these autoregressive coefficients were transformed into cepstral parameters[52,53]. 26 PLP coefficients (13 PLP coefficients from emotional speech signals + 13 PLP coefficients from emotional glottal signals)were derived for each frame and they were averaged over all frames.

#### Timbral texture features (TTFs)

Generally, timbral texture features were proposed for music-speech discrimination and speech recognition [54,55].The feature vector for describing timbral texture consists of the following features: spectral centriod, spectral flux, spectral rolloff, energy entropy, short-time energy and zero-crossing rate [54,55]. After obtaining the timbral texture features for each emotional speech and glottal signals, the following statistical parameters were computed such as standard deviation of timbral texture features, maximum by standard deviation of timbral texture features, maximum by median of timbral texture features, square of standard deviation by square of mean of timbral texture features. A total of 48 features (6 timbral texture features x 4 statistical features = 24 for each emotional speech signals + 6 timbral texture features x 4 statistical features = 24 for each glottal waveforms) were derived for each emotional speech signals and its glottal waveforms.

#### SWT based timbral texture features (SWT-TTFs)

The pre-emphasized emotional speech signals and glottal waveforms were decomposed into five levels using SWT with 10^th^order Daubechies wavelet. In this work, Daubechies wavelet has been chosen due to the following properties[56]: Time invariance, fast computation and sharp filter transition bands.Timbral texture features (Energy entropy, short-time energy, zero-crossing rate, spectral rolloff, spectral centriod and spectral flux) were extracted from the decomposed stationary wavelet coefficients (CA5, CD5, CD4, CD3, CD2 and CD1). After obtaining the timbral texture features for each decomposed stationary wavelet coefficients, the following statistical parameters were computed such as standard deviation of timbral texture features, maximum by standard deviation of timbral texture features, maximum by median of timbral texture features, square of standard deviation by square of mean of timbral texture features.A total of 288 features (6 timbral texture features x 4 statistical features x 6 subbands = 144 for each emotional speech signals + 6 timbral texture features x 4 statistical features x 6 subbands = 144 for each glottal waveforms) were derived for each emotional speech signals and its glottal waveforms after SWT decomposition.

#### Relative wavelet packet energy and entropy features (RWPFs)

The pre-emphasized emotional speech signals and glottal waveforms were segmented into 32 ms frames with 50% overlap. Each frame was decomposed into 4 levels using discrete wavelet packet transform with 10^th^order Daubechies waveletand relative wavelet packet energy and entropy features were derived for each of the decomposition nodes as given in the Equations (4) and (7).

$$EGY_{j,k}=\log_{10}\left({\frac{\sum \left|C_{j,k}\right|}{L}}^{2}\right)$$ (2)

$$EGY_{tot}=\sum EGY_{j,k}$$ (3)

$$\text{Relative wavelet packet energy},\text{RWPEGY}=\frac{EGY_{j,k}}{EGY_{tot}}$$ (4)

$$EPY_{j,k}=-{\sum \left|C_{j,k}\right|}^{2}\log_{10}{\left|C_{j,k}\right|}^{2}$$ (5)

$$EPY_{tot}=\sum EPY_{j,k}$$ (6)

$$\text{Relative wavelet packet entropy},\text{RWPEPY}=\frac{EPY_{j,k}}{EPY_{tot}}$$ (7)

where *j* = 1,2,3,…*m*, *k* = 0,1,2,…,2^*m*^-1, *m* is the number of decomposition level and *L* is the length of wavelet packet coefficients at each node(*j*,*k*). Four level wavelet packet decomposition give 30 wavelet packet nodes and features were extracted from all the nodes which yield 60 features (30 relative energy features + 30 relative entropy features). Similarly, the same features were extracted from emotional glottal signals. Finally, a total of 120 features were obtained. After obtaining 120 relative wavelet packet energy and entropy based features for each frame, they were averaged over all frames.

### PSO clustering for Feature Enhancement

Clustering methods have been widely used in various applications, such as statistics, software engineering, biology, psychology and other social sciences, in order to group the similar objects/instances in large amounts of data [57,58,59,60]. In any pattern recognition applications, escalating the inter-class variance and diminishing the intra-class variance of the attributes or features are the fundamental issues to improve the classification/recognition accuracy [57,58,59,60]. High intra-class variance and low inter-class variance among the features may degrade the performance of classifiers which results in poor emotion recognition rates. To decrease the intra-class variance and to increase the inter-class variance among the features, PSO based clustering was suggested in this work, to improve the discriminative ability of the extracted features. In 1995, Eberhart RC and Kennedy J have originally proposed a stochastic optimization approach which is called PSO [61]. The main problem with the PSO is that particles can get trapped in the local optimum. Van der Merwe D and Engelbrecht AP have suggested PSO for data clustering and obtained promising results[60]. Inspired by social interaction of humans in a global neighbourhood, Cohen SC and de Castro LN have proposed PSO based clustering to organize the data-points into clusters based on the interdependence of each particle [62]. In 2010, a modified PSO based clustering was proposed by Szabo, which did not require velocity and inertia weight during update procedure [58,59]. Mitchell Yuwono etal. have proposed a simple modification to mitigate the time complexity by reducing the frequency of distance matrix update [8]. Motivated by the previous works, PSO based clustering was suggested to enhance the discrimination abilityof the extracted features.The task of the PSO here is to search for the appropriate cluster centres such that the clustering metric (Euclidean distance) is minimized [6,8,58,59,60,62]. The steps involved in the PSO based clustering [6,8,58,59,60,62] are as follows:

Input: Feature Dateset *K*: number of classes(emotions)

Output: the location of *K* centroids (cluster centers)

 PSO_clustering(data, *K*)

 Generate the particles; each solution has its own K cluster centers

 selected randomly from dataset.

 For each particle

  Objective function = min(Euclidean distance)

*v*_*id*_ = *w* * *v*_*id*_ + *c*_1_ * *rand*(*p*_*id*_ - *x*_*id*_)+*c*_2_ * rand * (*p*_*gd*_ - *x*_*id*_)

*x*_*id*_ = *x*_*id*_ + *v*_*id*_

  Update *p*_*id*_

 End

  Update *p*_*gd*_

**Fig 2:**
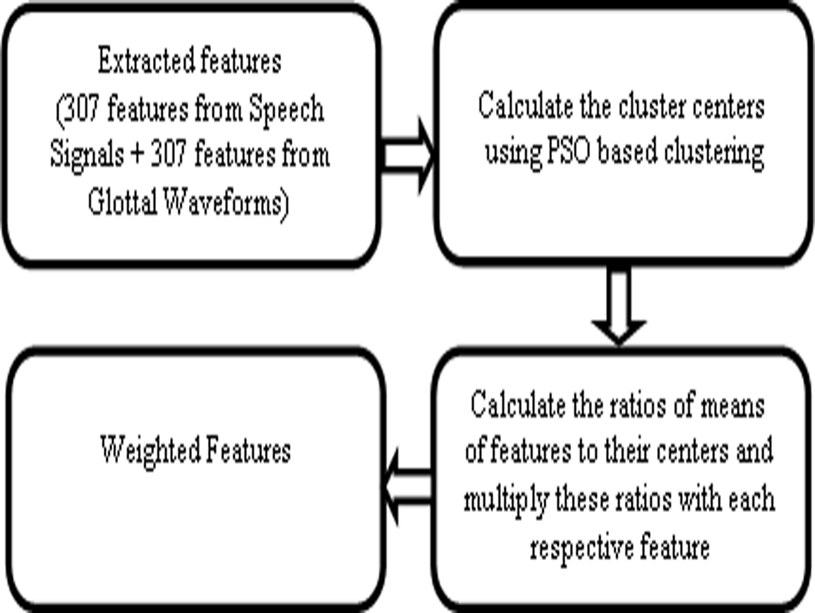
PSO based clustering for feature enhancement.

 End

where *w* is an inertia weight which plays an important role of balancing local and global search and usually decreased linearly [*w*(*t*+1) = 0.85*w(*t*)] during iterations [8]. *c*_*1*_ and *c*_*2*_ are two positive acceleration constants and fixed equally as 2.The initial value for *w* was fixed as 0.9 and maximum number of iterations was fixed as 100 [8]. If particles are getting trapped into local optimum, particles were reset to zero. Theworking of PSO based clustering as feature enhancement method is summarized (in Fig. 2) as follows: firstly, the appropriate cluster centersof each feature belonging to the dataset using PSO basedclustering were found. Next, the ratios of means of featuresto their respective cluster centers were calculated. Finally, these ratios weremultiplied with each respective feature to enhance their discriminative quality between the groups/classes.

### Feature Selection using PSO

**Fig 3:**
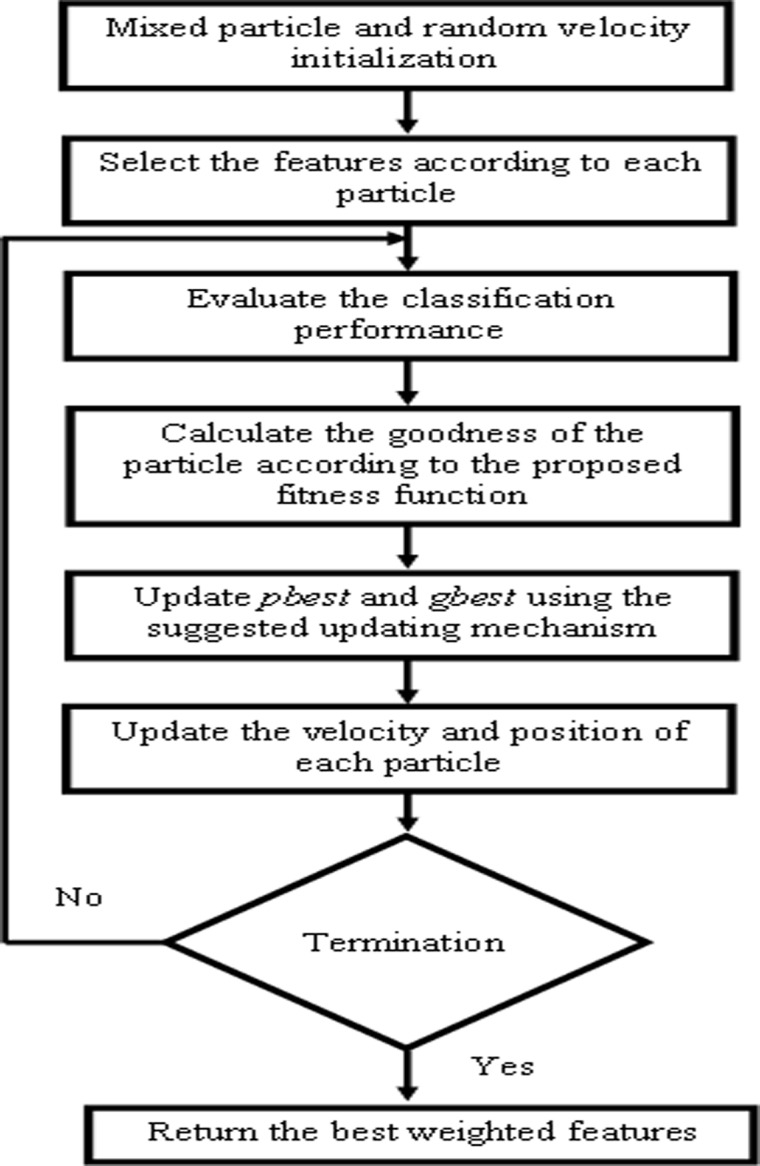
Process of Feature Selection using PSO.

Feature selection is an essential step prior to classification process to eliminate the redundant features, to select parsimonious, information-rich features and to avoid overfitting during classification [63,64,65,66]. Feature transformation and selection algorithms are commonly used to reduce the feature dimension and to select the most informative features. In this work, PSO based feature selection was proposed to select the best information-rich weighted features. The flowchart of the proposed PSO based feature selection was shown in Fig. 3. Conventionally, particles are initialized randomly. However, in this work, mixed initialization strategy was used. In this strategy, 50% of particles were initialized using a small number of features (10% of total features) and other particles were initialized using a large number of features (60% of total features) [11].

The main step in the PSO based feature selection is the goodness/fitness evaluation procedure. Generally, the two popular measures such as classification accuracy and error rate will be used in designing a fitness function. However, those measures will be unsuitable to measure the quality of the particles when dealing with the imbalanced dataset as they mislead the classification performance due to the emphasis on the influence of the majority class [67]. Hence, in this work, a new fitness function was developed to evaluate the fitness of the each particle, where the classification performance was evaluated through Geometric mean (G-mean).

$$Fitness=\;\alpha *(1-Gmean)\;+\;(1-\alpha )*\left(\frac{number\;of\;selected\;features}{All\;features}\right)$$ (8)

where *α* is used to show the relative importance of the classification performance (G-mean) and (1-*α*) shows the relative importance of the number of features. As the classification performance is more important than the number of features, the value for *α* was fixed as 0.8. Based on the fitness function (Equation 8), the quality of each particle was calculated. After evaluating the fitness of all particles, the algorithm updates the *pbest* and *gbest*, and then updates the velocity and position of each particle. *pbest* and *gbest*were updated in two situations. In first situation, the current *pbest*was updated, if the classification performance (G-mean) of the particle’snew position was better than that of previous *pbest* and the number of featureswas not larger than previous *pbest*. In second situation, the current *pbest* is updated, if thenumber of features was smaller than previous *pbest* and the classification performance (G-mean) of the new position was the same or better than the current *pbest*. *gbest*was updated in the same way [11]. The position of a particle represents a selected feature subset. In our binaryPSO, *v*-shaped transfer function was applied to transform the velocity fromcontinuous space to probability space [9]:

$$S\left(x_{i}^{k}\left(t\right)\right)\;=\;\frac{2}{\pi }arc\;\tan\left(\frac{\pi }{2}x_{i}^{k}\left(t\right)\right)$$ (9)

$$v_{i}^{k}\left(t\right)\;=\;w*v_{i}^{i}\left(t\right)+c_{1}*rand*\left(pbest_{i}^{k}\left(t\right)-x_{i}^{k}\left(t\right)\right)+c_{2}*rand*\left(gbest_{i}^{k}\left(t\right)-x_{i}^{k}\left(t\right)\right)\;$$ (10)

As we have used *v*-shaped transfer function, the following position updating rules should be used [9].

xik(t+1) = {x~ik(t)rand < T(vik(t+1))xik(t)rand≥ T(vik(t+1)) (11)

The PSO simulation will stop when a pre-defined stopping criterion, e.,g the maximum number of iterations or an optimal fitness value, has been reached. Maximum number of iterations was fixed as 100. If particles are getting trapped into local optimum, particles were reset to zero.

The initial value of *w* was set as 1.4 and changed adaptively during iteration using the following equation [68].

$$w\;=\;\left(w-0.4\right)\;*\left(t_{\max}-t\right)/\left(t_{\max}+0.4\right)$$ (12)

where *t*_*max*_ and *t* are the maximum number of iterations and the current iteration.

### Extreme Learning Machine

A new learning algorithm for the single hidden layer feedforward networks(SLFNs) called as ELM was proposed by G.B. Huang et.al [69,70,71,72]. It has been widely used in various applications to overcome the slow training speed and over-fitting problems of the conventional neural network learning algorithms [69,70,71,72]. The brief idea of ELM is given as follows:[69,70,71,72]

For the given *N* training samples, the output of a SLFN network with *L* hidden nodes can be expressed as the following:

$$f_{L}\left(x_{j}\right)\;=\;\sum_{i}^{L}\beta_{i}g\left(w_{i}.x_{j}+b_{i}\right),j=1,2,3,\ldots ,N$$ (13)

It can be written as *f*(*x*) = *h*(*x*) *β*, where *x*_*j*_,*w*_*i*_ and *b*_*i*_ are the input training vector, input weights and biases to the hidden layer respectively. *β*_*i*_ is the output weights that links the *i*-th hidden node to the output layer and *g*(.) is the activation function of the hidden nodes. Training an SLFN is simply finding a least-square solution by using Moore-Penrose generalized inverse:

$$\hat{\beta }\;=\;H†T,$$ (14)

Where *H†* = (*H’H*)^-1^ *H’* or *H’*(*HH’*)^-1^, depending on the singularity of *H’H* or *HH’*. Assume that *H’H* is not a singular, the coefficient 1/*ε* (*ε* is positive regularization coefficient) is added to the diagonal of *H’H* in the calculation of the output weights *β*_*i*_. Hence, more stable learning system with better generalization performance can be obtained.

The output function of ELM can be written compactly as

$$f\left(x\right)\;=\;h\left(x\right)\;H'{\left(\frac{1}{\varepsilon }+HH'\right)}^{-1}T$$ (15)

In this ELM kernel implementation, the hidden layer feature mappings need not to be known to users and Gaussian kernel was used. Best values for positive regularization coefficient (*ε*) and Gaussian kernel parameter were found empirically after several experiments.

### Emotion Recognition Results

**Fig 4:**
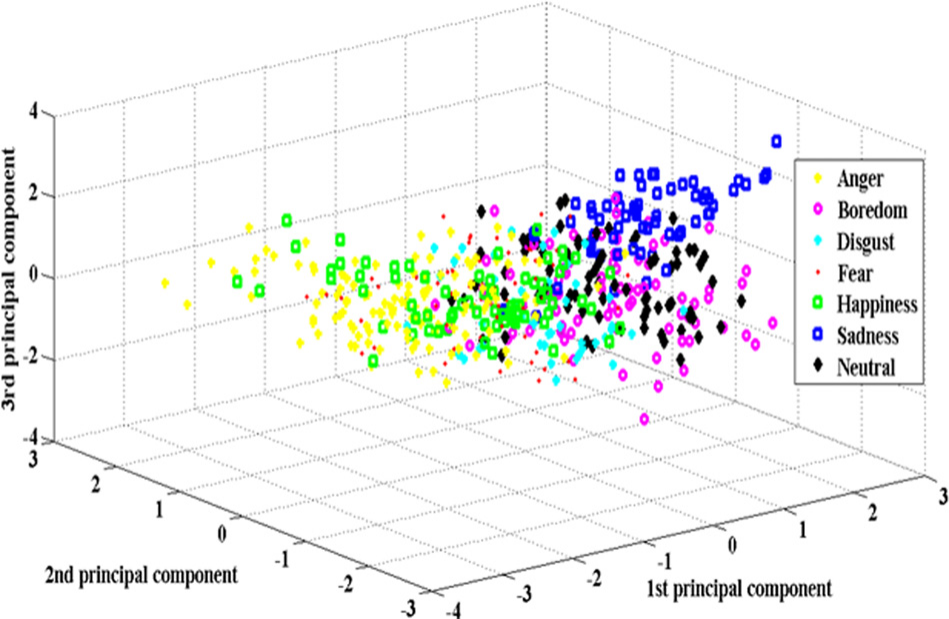
Class distribution plots of raw features.

**Fig 5:**
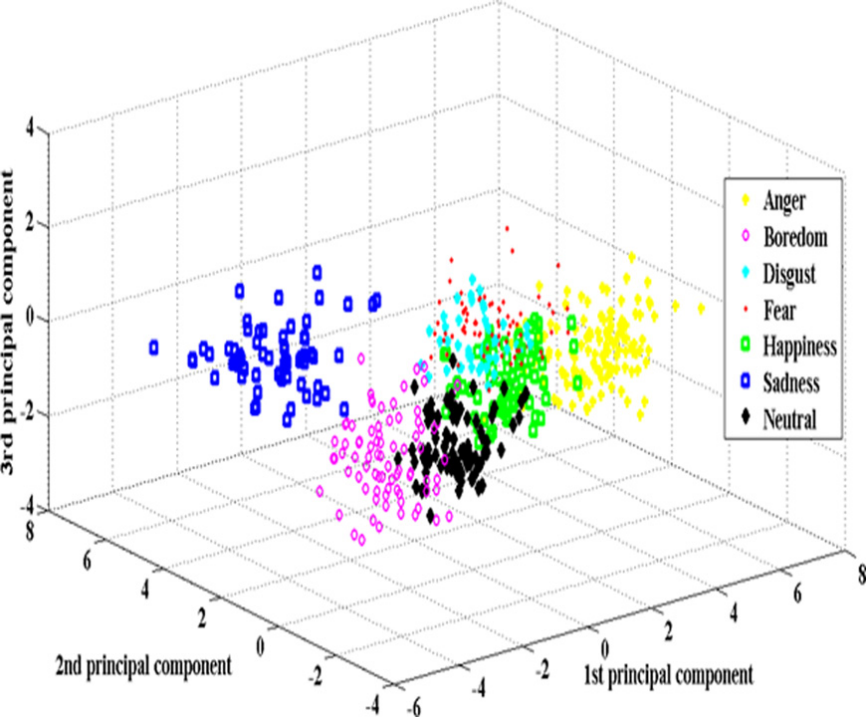
Class distribution plots of weighted features.

From the literature, it can be observed that the high emotion recognition rates can be achieved for the recognition between high-activation emotions and low-activation emotions; however, recognition between different emotions (multi-class) is still challenging. To improve the speaker-independent emotion recognition accuracy, we have suggested PSO based feature enhancement and feature selection method. In addition to speaker-independent (SI) emotion recognition, we have also conducted experiments on speaker-dependent (SD), gender dependent (GD-male and GD-female) environments. Three different emotional speech databases were used to gauge the robustness of the proposed method. From the speech utterances, glottal waveforms were derived. A total of 614 features derived from both speech utterances (307 features) and glottal waveforms (307 features). PSO based clustering was used to enhance the discriminative ability of the extracted features and PSO based feature selection was proposed to select the best weighted features. Modified particle initialization, *pbest* and *gbest* update scheme and a new fitness function were used to improve the feature selection process. ELM kernel classifier was used. The proposed method was implemented under MATLAB platform using a LAPTOP with Intel Core i7–2.2 GHz and 4 GB RAM. Figs. 4 and 5 depicts the class distribution plots of raw and weighted features for BES database. From the Fig. 4, a higher degree of overlap among raw features can be observed. According to the Fig. 5, inferences show that after PSO based feature enhancement, the weighted features could provide relatively better separable class distribution.

Twenty five independent simulations (runs) of PSO based clustering and PSO based feature selection were conducted. Table 2 provides the details of selected weighted features using the proposed wrapper based PSO. Most frequently selected weighted features were identified during twenty-five independent PSO runs and used for emotion recognition experiments.

**Table 2:** List of number of selected weighted features.

List of Features	BES		SES		SAVEE	
	From Speech Signals	From Glottal Signals	From Speech Signals	From Glottal Signals	From Speech Signals	From Glottal Signals
MFCCs (24+24)	1	1	2	3	3	4
LPCCs (18+18)	3	2	3	2	2	2
GTFBOs (24+24)	2	1	3	3	2	3
PLPs (13+13)	1	1	2	1	2	1
TTFs (24+24)	3	2	3	2	3	1
SWTTTFs (144+144)	8	10	9	10	14	11
RWPFs (60+60)	5	5	4	4	1	4
Total Selected Features (average)	23	22	26	25	27	26
Total Selected Features (Minimum)	16	18	16	18	21	17
Total Selected Features (Maximum)	28	29	33	31	36	32

Table 3, 4 and 5 shows the average emotion recognition results in terms of confusion matrices for raw, weighed and selected weighted features under different experiments.

**Table 3:** Confusion matrices for emotion recognition using raw, weighted and selected weighted features (BES).

Experiments	Emo-tions	Raw Features							Weighted Features							Selected Weighted Features						
		Ang	Bor	Dis	Fea	Hap	Sad	Neu	Ang	Bor	Dis	Fea	Hap	Sad	Neu	Ang	Bor	Dis	Fea	Hap	Sad	Neu
**SD**	**Ang**	**94.80**	0.00	0.87	0.34	3.99	0.00	0.00	**100**	0.00	0.00	0.00	0.00	0.00	0.00	**98.87**	0.00	0.15	0.37	3.59	0.00	0.00
	**Bor**	0.00	**80.54**	1.68	1.06	0.00	2.80	13.92	0.00	**100**	0.00	0.00	0.00	0.00	0.00	0.00	**98.28**	0.64	0.00	0.00	0.03	2.49
	**Dis**	4.87	1.25	**79.55**	8.01	0.00	0.00	6.32	0.00	0.00	**100**	0.00	0.00	0.00	0.00	0.06	0.00	**97.99**	0.71	0.41	0.03	0.00
	**Fea**	3.37	1.11	1.96	**87.17**	2.97	2.65	0.77	0.00	0.00	0.80	**99.20**	0.00	0.00	0.00	0.16	0.33	0.37	**97.27**	0.70	0.20	0.19
	**Hap**	23.27	0.00	4.32	6.75	**62.39**	1.00	2.27	0.00	0.00	0.00	0.00	**100**	0.00	0.00	0.90	0.00	0.51	1.19	**94.92**	0.00	0.40
	**Sad**	0.00	3.73	0.00	1.97	0.00	**91.94**	2.37	0.00	0.00	0.00	0.00	0.00	**100**	0.00	0.00	0.03	0.00	0.34	0.00	**99.17**	0.16
	**Neu**	0.67	7.61	0.00	0.00	0.00	5.23	**86.50**	0.00	0.00	0.00	0.00	0.00	0.00	**100**	0.00	1.36	0.34	0.13	0.38	0.57	**96.77**
**SI**	**Ang**	**88.68**	0.00	2.51	1.74	7.07	0.00	0.00	**99.65**	0.00	0.00	0.00	0.35	0.00	0.00	**98.46**	0.00	0.49	1.15	5.78	0.00	0.00
	**Bor**	0.00	**70.94**	3.07	1.98	0.00	8.79	15.21	0.00	**100**	0.00	0.00	0.00	0.00	0.00	0.00	**97.55**	1.95	0.00	0.10	0.29	3.73
	**Dis**	10.69	21.25	**48.89**	8.22	2.00	0.00	8.94	0.00	0.44	**99.56**	0.00	0.00	0.00	0.00	0.03	0.16	**95.22**	0.38	1.09	0.60	0.04
	**Fea**	12.62	5.91	5.05	**64.92**	7.05	3.40	1.05	0.27	0.00	0.00	**99.73**	0.00	0.00	0.00	0.16	0.55	0.77	**97.16**	1.08	0.79	0.88
	**Hap**	37.66	1.11	3.61	12.33	**41.07**	0.00	4.22	1.34	0.00	0.00	0.00	**98.66**	0.00	0.00	1.35	0.00	0.66	0.74	**91.58**	0.00	0.36
	**Sad**	0.00	8.50	3.08	7.18	0.00	**79.99**	1.25	0.00	0.00	0.00	0.92	0.00	**99.08**	0.00	0.00	0.12	0.00	0.40	0.00	**97.67**	0.35
	**Neu**	1.25	16.02	6.53	3.08	0.95	3.47	**68.70**	0.00	0.56	0.00	0.00	0.00	0.00	**99.44**	0.00	1.62	0.89	0.17	0.36	0.65	**94.65**
**GD (Male)**	**Ang**	**94.17**	0.00	0.83	0.83	4.17	0.00	0.00	**100**	0.00	0.00	0.00	0.00	0.00	0.00	**99.47**	0.00	1.00	0.11	4.24	0.00	0.00
	**Bor**	0.00	**61.43**	0.00	2.86	0.00	8.57	27.14	0.00	**100**	0.00	0.00	0.00	0.00	0.00	0.00	**97.94**	1.20	0.00	0.00	0.00	1.25
	**Dis**	10.00	5.00	**55.00**	25.00	0.00	0.00	5.00	0.00	0.00	**100**	0.00	0.00	0.00	0.00	0.00	0.00	**85.60**	0.34	0.00	0.00	0.00
	**Fea**	2.86	0.00	0.00	**92.86**	1.43	0.00	2.86	0.00	0.00	0.00	**100**	0.00	0.00	0.00	0.17	0.34	3.60	**98.57**	0.64	0.16	0.20
	**Hap**	20.00	0.00	0.00	8.00	**72.00**	0.00	0.00	0.00	0.00	0.00	0.00	**100**	0.00	0.00	0.37	0.06	3.60	0.52	**95.04**	0.00	0.35
	**Sad**	0.00	10.00	0.00	0.00	0.00	**88.00**	2.00	0.00	0.00	0.00	0.00	0.00	**100**	0.00	0.00	0.12	0.20	0.34	0.00	**98.96**	0.30
	**Neu**	1.25	3.75	0.00	7.50	1.25	1.25	**85.00**	0.00	0.00	0.00	0.00	0.00	0.00	**100**	0.00	1.54	4.80	0.12	0.08	0.88	**97.90**
**GD (Female)**	**Ang**	**96.92**	0.00	0.77	0.00	2.31	0.00	0.00	**100**	0.00	0.00	0.00	0.00	0.00	0.00	**98.71**	0.00	0.29	0.40	4.13	0.00	0.00
	**Bor**	0.00	**88.89**	0.00	0.00	0.00	0.00	11.11	0.00	**100**	0.00	0.00	0.00	0.00	0.00	0.00	**98.67**	0.00	0.06	0.00	0.00	3.60
	**Dis**	8.57	0.00	**85.71**	5.71	0.00	0.00	0.00	0.00	0.00	**100**	0.00	0.00	0.00	0.00	0.19	0.00	**99.09**	1.71	0.31	0.06	0.00
	**Fea**	2.86	0.00	2.86	**87.14**	7.14	0.00	0.00	0.00	0.00	2.00	**98.00**	0.00	0.00	0.00	0.12	0.09	0.34	**96.80**	0.53	0.06	0.15
	**Hap**	33.33	0.00	0.00	1.11	**60.00**	2.22	3.33	0.00	0.00	0.00	0.00	**100**	0.00	0.00	0.98	0.00	0.29	0.97	**94.76**	0.00	0.05
	**Sad**	0.00	0.00	1.43	0.00	0.00	**98.57**	0.00	0.00	0.00	0.00	0.00	0.00	**100**	0.00	0.00	0.00	0.00	0.00	0.00	**99.77**	0.00
	**Neu**	0.00	13.75	0.00	0.00	0.00	0.00	**86.25**	0.00	0.50	0.00	0.00	0.00	0.00	**99.50**	0.00	1.24	0.00	0.06	0.27	0.11	**96.20**

**Table 4:** Confusion matrices for emotion recognition using raw, weighted and selected weighted features (SES).

Experiments	Emotions	Raw Features					Weighted Features					Selected Weighted Features				
		Neu	Sur	Ang	Sad	Hap	Ang	Dis	Fea	Hap	Neu	Ang	Dis	Fea	Hap	Neu
**SD**	**Neu**	**45.21**	6.04	11.04	24.17	13.54	**91.63**	2.29	2.21	2.21	1.67	**83.47**	5.32	2.44	3.33	3.13
	**Sur**	10.21	**44.17**	12.08	16.25	17.29	2.08	**93.08**	1.67	1.38	1.79	4.73	**82.23**	3.35	4.02	2.03
	**Ang**	9.17	10.21	**60.83**	6.25	13.54	0.42	0.21	**95.54**	1.00	2.83	3.92	5.39	**85.54**	4.39	5.03
	**Sad**	16.46	8.33	7.08	**62.92**	5.21	1.13	0.33	2.38	**94.63**	1.54	3.43	2.88	2.53	**81.54**	3.71
	**Hap**	12.92	18.75	17.50	6.25	**44.58**	0.29	0.04	1.46	0.33	**97.88**	4.44	4.18	6.13	6.72	**86.10**
**SI**	**Neu**	**32.50**	20.83	15.00	16.67	15.00	**88.00**	3.25	2.75	3.42	2.58	**73.53**	10.52	5.07	4.85	7.95
	**Sur**	21.25	**29.17**	16.25	13.33	20.00	4.75	**82.42**	5.33	4.92	2.58	9.05	**65.55**	6.25	6.02	3.88
	**Ang**	21.67	23.75	**31.25**	6.25	17.08	1.75	1.75	**82.00**	5.33	9.17	5.61	10.70	**71.73**	7.46	9.77
	**Sad**	37.08	23.75	7.50	**25.00**	6.67	0.08	2.50	5.67	**89.25**	2.50	5.43	7.83	6.97	**72.78**	8.10
	**Hap**	20.42	25.42	17.92	8.33	**27.92**	3.33	0.83	5.17	2.58	**88.08**	6.36	5.40	9.98	8.88	**70.30**
GD (Male)	**Neu**	**67.50**	0.00	9.58	0.00	22.92	**95.50**	0.33	1.83	1.83	0.50	**89.87**	0.60	4.07	1.73	0.88
	**Sur**	0.00	**77.08**	0.00	22.92	0.00	0.00	**99.75**	0.00	0.25	0.00	0.55	**98.68**	0.23	1.53	0.25
	**Ang**	7.92	0.00	**75.00**	0.00	17.08	2.08	0.00	**93.58**	0.25	4.08	4.80	0.08	**88.08**	0.77	3.60
	**Sad**	0.00	15.83	0.00	**84.17**	0.00	0.50	0.75	0.08	**98.25**	0.42	2.83	0.35	1.00	**93.93**	1.68
	**Hap**	22.08	0.00	21.25	0.00	**56.67**	0.00	0.00	1.75	0.25	**98.00**	1.95	0.29	6.62	2.03	**93.58**
GD (Female)	**Neu**	**62.50**	0.42	23.33	0.00	13.75	**99.08**	0.00	0.33	0.50	0.08	**98.98**	0.57	0.63	1.85	0.32
	**Sur**	0.00	**79.58**	0.42	20.00	0.00	1.75	**90.33**	0.83	2.58	4.50	0.17	**89.82**	0.58	3.83	1.72
	**Ang**	25.42	0.00	**57.50**	0.00	17.08	0.00	0.08	**99.83**	0.08	0.00	0.20	1.22	**98.02**	0.82	0.78
	**Sad**	1.25	22.92	0.00	**75.83**	0.00	1.67	0.25	0.83	**96.25**	1.00	0.40	4.37	0.39	**90.37**	2.58
	**Hap**	17.50	0.00	17.08	0.00	**65.42**	0.00	0.17	0.00	0.83	**99.00**	0.25	4.03	0.38	3.13	**94.60**

**Table 5:** Confusion matrices for emotion recognition using raw, weighted and selected weighted features (SAVEE).

Experiments	Emotions	Raw Features							Weighted Features							Selected Weighted Features						
		Ang	Dis	Fea	Hap	Neu	Sad	Sur	Ang	Dis	Fea	Hap	Neu	Sad	Sur	Ang	Dis	Fea	Hap	Neu	Sad	Sur
**SD/GD**	**Ang**	**77.50**	0.83	0.83	10.00	2.50	0.00	8.33	**98.83**	0.00	0.00	1.17	0.00	0.00	0.00	**95.93**	0.73	0.17	2.90	0.03	0.00	0.30
	**Dis**	3.33	**60.00**	6.67	0.83	21.67	4.17	3.33	0.00	**97.83**	0.00	0.00	2.17	0.00	0.00	0.30	**90.53**	1.20	1.17	0.67	1.33	0.43
	**Fea**	4.17	7.50	**62.50**	9.17	2.50	1.67	12.50	0.00	0.00	**99.33**	0.00	0.00	0.00	0.67	0.27	0.67	**91.67**	1.17	0.20	0.27	1.60
	**Hap**	5.00	4.17	4.17	**70.00**	0.00	0.83	15.83	0.83	0.00	0.00	**98.17**	0.00	0.00	1.00	3.03	0.73	1.70	**92.17**	0.02	0.03	4.10
	**Neu**	1.25	2.08	3.33	0.42	**88.75**	2.50	1.67	0.00	0.00	0.00	0.00	**99.58**	0.42	0.00	0.07	5.83	0.33	0.00	**98.47**	2.16	0.17
	**Sad**	1.67	4.17	2.50	0.00	2.50	**88.33**	0.83	0.00	0.17	0.00	0.00	0.17	**99.67**	0.00	0.00	1.07	0.50	0.10	0.52	**96.00**	0.13
	**Sur**	0.83	5.00	15.83	17.50	0.83	0.83	**59.17**	0.00	0.00	0.17	0.50	0.00	0.00	**99.33**	0.40	0.43	4.43	2.50	0.10	0.20	**93.27**
**SI**	**Ang**	**55.00**	20.00	0.00	3.33	15.00	0.00	6.67	**67.33**	5.33	0.00	13.00	6.00	0.00	8.33	**69.07**	0.93	0.40	17.00	0.30	0.60	1.27
	**Dis**	10.00	**23.33**	3.33	5.00	41.67	13.33	3.33	0.00	**83.00**	1.33	0.33	13.67	1.67	0.00	5.93	**69.07**	5.40	4.40	11.93	7.20	4.67
	**Fea**	18.33	25.00	**15.00**	0.00	11.67	8.33	21.67	0.00	5.00	**72.67**	7.00	2.67	2.00	10.67	1.07	4.07	**67.27**	5.73	0.10	3.20	9.13
	**Hap**	28.33	21.67	3.33	**15.00**	8.33	0.00	23.33	11.67	4.33	4.00	**67.33**	1.33	0.00	11.33	9.47	1.93	6.47	**55.93**	0.43	1.27	11.67
	**Neu**	0.00	23.33	0.00	0.00	**73.33**	0.00	3.33	0.00	11.33	0.00	0.17	**86.50**	2.00	0.00	4.13	13.67	1.67	2.27	**79.27**	12.87	0.53
	**Sad**	0.00	40.00	1.67	0.00	21.67	**36.67**	0.00	0.00	15.00	0.00	0.33	8.33	**76.33**	0.00	1.87	6.13	5.47	2.00	5.40	**72.60**	2.00
	**Sur**	15.00	28.33	10.00	11.67	8.33	0.00	**26.67**	0.00	5.00	7.00	13.33	0.33	0.00	**74.33**	8.47	4.20	13.33	12.67	2.57	2.27	**70.73**

According to the Table 3 (BES database), the average recognition rates (seven emotions) using all the weighted features were significantly improved from 83.27% (SD), 66.17% (SI), 78.35% (GD-Male) 86.21% (GD-Female) to 99.89% (SD), 99.45% (SI), 100% (GD-Male), 99.64% (GD-Female) under different experiments. Using the best weighted features (average of 23 weighted features from speech signals + average of 22 weighted features from glottal waveforms), 97.61% (SD), 96.04% (SI), 96.21% (GD-Male), 97.71% (GD-Female) were achieved.

From Table 4 (SES database), it can be observed that the average recognition rates (five emotions) using all the weighted features were significantly increased from 51.54% (SD), 29.17% (SI), 72.08% (GD-Male), 68.17% (GD-Female) to 94.55% (SD), 85.95% (SI), 97.02% (GD-Male), 96.90% (GD-Female). After selecting the best weighted features (average of 26 weighted features from speech signals + average of 25 weighted features from glottal waveforms), 83.78% (SD), 70.78% (SI), 92.83% (GD-Male), 94.36% (GD-Female) were achieved. For SAVEE database (Table 5), average emotion recognition rates (seven emotions) using all the weighted features were improved from 72.32% (SD/GD), 35.00% (SI) to 98.96% (SD/GD), 75.36% (SI). An average emotion recognition rate of 94.01% in SD experiment and 69.13% in SI experiment were achieved using the best weighted features (average of 27 weighted features from speech signals + average of 26 weighted features from glottal waveforms). The results obtained for BES and SAVEE database were significantly better than the results presented in the literature. A paired t-test was performed with the significance level of 0.05 on the emotion recognition results obtained using the raw and weighted features. In almost all cases, emotion recognition results obtained using the weighted features were significantly better than using the raw features. From the above experiments and results, higher emotion recognition rates between different emotions were obtained using weighted features compared to raw features.

## Conclusions

Improved speaker-independent multi-class emotion recognition can provide a better communication between human and machine. In this study, we have investigated the effectiveness of PSO based clustering and feature selection algorithm to enhance the extracted speech features and to improve the multi-class speaker independent emotion recognition accuracy as well. Emotion recognition experiments have been conducted with three different emotional speech databases using the proposed method. Both speech and glottal waveforms were subjected to feature extraction. Four different experiments such as SD, SI, GD-Male and GD-Female were conducted. After PSO based clustering, the discrimination ability of the extracted features has been improved which provides higher emotion recognition accuracy. Only less than 10% of total weighted features have been selected based on PSO based feature selection with improved fitness function. The experimental results demonstrated the merits of the proposed method in the field of emotion recognition. The highest emotion recognition accuracy in all experiments also showed the effectiveness of the ELM-kernel classifier. From the results, we can also conclude that the proposed method yielded a higher emotion recognition accuracy compared to the state of the art works in the literature for the emotional speech databases under test. In future work, the results of proposed PSO based clustering and feature selection will be compared with other counterparts. The proposed method will be tested using larger corpora and more naturalistic corpora. Cross-cultural or cross-linguistic validity of the proposed method will also be performed.
